# 1922. Outbreak and Management of COVID-19 and Infection Prevention Control Practices at a Community Living Center in Veterans Administration Hospital, North Texas

**DOI:** 10.1093/ofid/ofac492.1549

**Published:** 2022-12-15

**Authors:** John J Hanna, Denisse Silva-Rodriguez, Angela Christie-Smith, Andrew O Psenicka, Sherry R Reid, Ikwo K Oboho

**Affiliations:** University of Texas Southwestern, Dallas, Texas; VA North Texas Health Care System, Forney, Texas; Veterans Affairs, Bonham, Texas; VA North Texas Health Care System, Forney, Texas; Veterans Affairs North Texas Health Care System, Dallas, Texas; VA North Texas Healthcare System, UT Southwestern Medical Center Dallas, TX, Dallas, TX

## Abstract

**Background:**

The increase in SARS-CoV-2 cases due to the omicron wave led to significant utilization of healthcare resources and reduced acute care hospital beds at the Veterans Administration Hospital, North Texas Health Care System (VANTHCS). As a result, veterans with non-severe disease were managed at a VANTHCS community living center (CLC) during a COVID-19 outbreak.

**Methods:**

Veterans residing at the CLC with laboratory-confirmed cases of SARS-CoV-2 (the virus that causes COVID-19) by polymerase chain reaction diagnosed from January 1 to February 15, 2022, were included in the descriptive analysis. We described resident characteristics and outcomes and infection control practices (IPC) implemented to control the outbreak. Resident data was ascertained from the COVID-19 facility dashboard and medical record system.

**Results:**

From January 1–February 15, 2022, 33 adults residing at the CLC were diagnosed COVID-19. Most infections (93.9%) occurred between January 12–24 (figure 1). The median age was 76 years [interquartile range, 71–80 years] and 30 (90.9%) were men and 25 (75.8%) were white and 5 (15.2%) African American (table 1). Among the total cases, 9 (27.3%) resided in the dementia unit. Nineteen of 33 (57.6%) were asymptomatic. Overall, 28 (84.8%) were documented to be fully vaccinated against SARS-CoV-2 and 24 (72.7%) were boosted. Obesity, ischemic heart disease, chronic obstructive pulmonary disease, and stroke were the most common comorbidities. Residents were cohorted based on COVID-19 results. A multidisciplinary team was convened, and staff were fit tested for appropriate personal protective equipment (PPE) and received refresher training on hand hygiene, donning and doffing of PPE. Most residents were determined to have mild or moderate COVID-19 and managed at the CLC while 7 (21.2%) were hospitalized in the acute care hospital. For management of COVID-19, 11 (33.3%) received dexamethasone and 25 (75.8%) received remdesivir. Overall, 32 (97%) residents survived while one hospice resident was transferred to acute care and died; only 1 resident required ICU admission.

Epidemic curve of laboratory-confirmed coronavirus disease 2019 (COVID-19) disease at a Community Living Center, Veterans Administration Hospital, North Texas Health Care System, January–February 2022.

**Figure:**
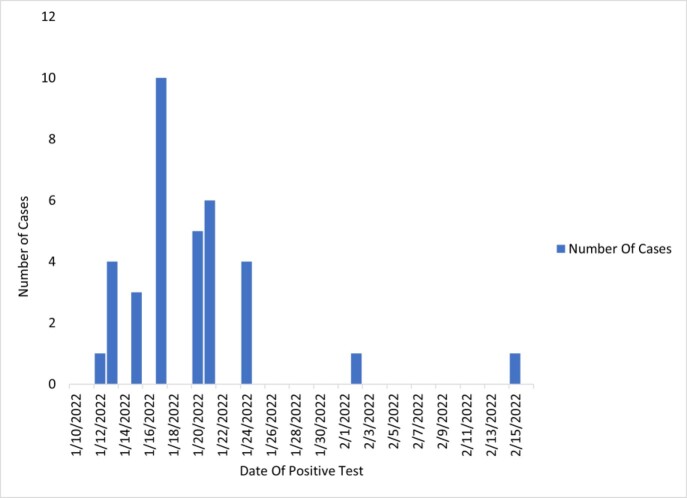

**Table 1:**
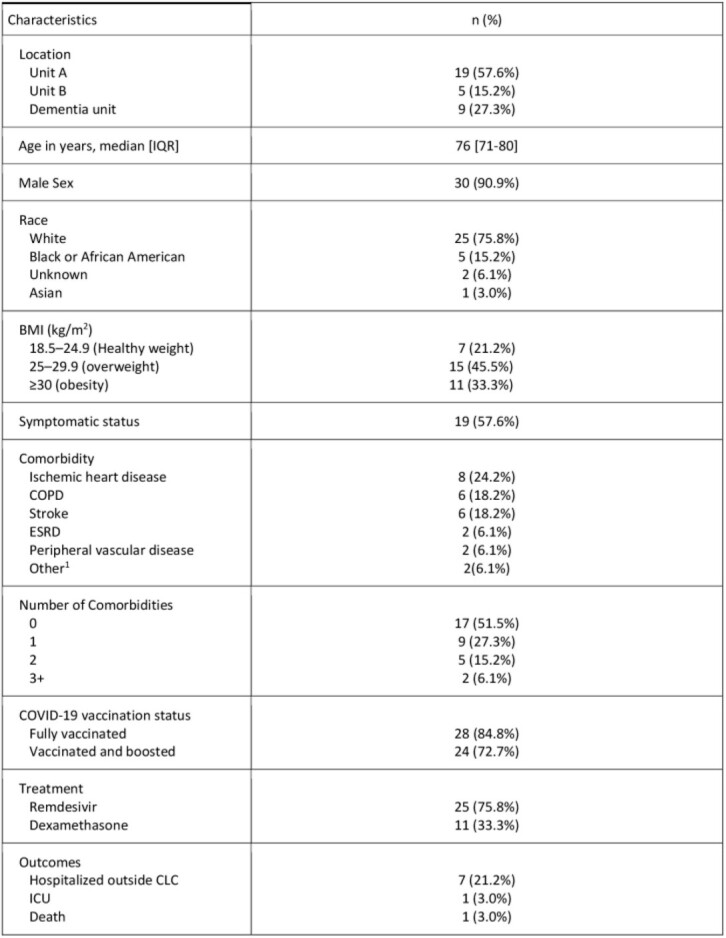
Epidemiological Characteristics, and Outcomes of Laboratory-confirmed COVID-19 cases (N=33)

**Conclusion:**

It is feasible to administer COVID-19 therapies to high-risk residents with mild-moderate disease in a CLC with a multidisciplinary team and IPC strategies.

**Disclosures:**

**All Authors**: No reported disclosures.

**Table 1:** Epidemiological Characteristics, and Outcomes of Laboratory-confirmed COVID-19 cases (N=33)

Abbreviation: BMI, body mass index; COPD, Chronic Obstructive Pulmonary Disease; ESRD, end stage renal disease

1Other comorbidity (asthma n=1 and chronic liver disease n=2)

